# Collagen–based scaffolds for skin tissue engineering

**Published:** 2011-05-25

**Authors:** A Gaspar, L Moldovan, D Constantin, AM Stanciuc, PM Sarbu Boeti, IC Efrimescu

**Affiliations:** * National Institute of Research and Development for Biological Sciences, BucharestRomania; ** Fundeni Clinical Institute, BucharestRomania

**Keywords:** tissue engineering, collagen, agarose, biostability, biocompatibility

## Abstract

The aim of this study was to obtain four collagen based porous scaffolds and to assess their in vitro biocompatibility and biodegradability in order to use them for skin tissue engineering. We have prepared four variants of collagen–based biodegradable sponges by liophilization of type I collagen solution and three variants of collagen–agarose mixture in different ratios 2:1 (A), 1:1 (B) and 1:2 (C). These scaffolds had microporous structure with a higher than 98% porosity and a reduced biodegradation after their exposure to UV radiation. The incorporation of agarose into the collagen scaffolds has improved their structural stability. In vitro biocompatibility testing for the four types of sponges was performed on a stabilized fibroblast cell line and showed that both cell viability and morphology were not altered by collagen and collagen–agarose variants A and B sponges. These three porous sponges demonstrated potential for future application as cell scaffolds in skin tissue engineering.

## Introduction

Tissue engineering is a new emerging biotechnology that focuses on the synthesis of new 3–D biofunctional materials to serve as porous scaffolds for cell attachment. These constructs, built from synthetic or natural polymers, can be used to produce neo–tissue with mature extracellular matrix and to guide the proliferation and spread of seeded cells in vitro and in vivo. The main requirements for skin biomaterials are biocompatibility, degradability and structural integrity. Collagen (COL) is a natural polymer abundant in all vertebrates, which provides the major mechanical support for cell attachment. It is a biomaterial of interest to the medical community due to its advantageous properties that recommend it for tissue engineering [1]. These properties are conferred by the COL molecule's native structure and chemical composition. Many types of COL have been discovered, which differ in their three–dimensional structure and their amino acid sequence, in order to meet the functional needs of different tissues [2]. In recent years, special attention was paid to COL, due to its excellent biocompatibility and its ability to degrade into well–tolerated compounds. The favorable influence of COL on cell infiltration and wound healing are demonstrated in previous studies [3–9]. Applications range from treating medical conditions such as nasal bleeding, burns, tablets for weight control, cosmetics but also light tissue defects, plastic surgery and even collagen gels combined with chemotherapic agents for cancer treatment [10]. 

The aim of this study was to obtain and characterize the four COL based porous scaffolds and to assess their in vitro biocompatibility and physico–chemical properties. Our work investigated the effect of agarose (AG) over the COL based scaffolds' biostability and in vitro biocompatibility by using a mouse fibroblast cell line in order to establish if these supports can be used for skin tissue engineering.

## Materials and methods

### Sponge preparation

An 8% of COL type I solution, obtained from enzymatic extraction from the bovine tendon, was mixed with the 1% AG (Sigma) at 370C, stirring continuously by using a blender. Four types of solutions were prepared, collagen type I , COL–AG 2:1 (A), COL–AG 1:1 (B) and COL–AG 1:2 (C). In order to obtain solid support these solutions were lyophilized by using a Gamma 1–16 LSC, Christ liofilizator (frozen temperature was –40 degrees C). The freeze–dried materials were exposed to UV radiation, for 8h, in an UV sterilization cabinet (Scie–Plas, England).

### Density and porosity measurement

The density (d) and porosity (e) of COL and COL–AG scaffolds were measured by using the water displacement method [11]. Briefly, a sample with a known weight (w) was immersed into a graded test tube having a known volume of water (v1). The sample was kept in water for 30 min and pressed, to force air from the scaffold and allow the water to penetrate and fill the pores. The total volume of water plus the water–impregnated sponge was recorded as v2. The water–impregnated scaffold was removed from the test tube and the residual water volume was recorded as v3. The following equations were used:

d = w/(v2–v3)  (1)    and      

e = (ν1–ν3)/( ν2–ν3)x 100   (2)

Three measurements were taken for each average value.

### In vitro degradation test

This test was performed by using bacterial collagenase. Briefly,  UV–treated  (8 hours) and untreated scaffolds of about 5 mg dry weight were incubated in 0.1 M Tris–HCl (pH 7.4) containing 2 U/mL bacterial collagenase (Clostridium histolyticum, EC 3.4.24.3, Sigma Chemical Co.), at 370C. After 24h, the reaction was stopped and the extent of scaffold degradation was determined by measuring the amount of protein in the supernatant. Biodegradability was calculated in comparison with the control sample (untreated COL scaffold) considered to be 100% degraded. The experiments were performed in triplicate.

### Scaffold effect on cell culture

- Cell culture:All biocompatibility assays were performed by using the NCTC cell line (clone L929), acquired from ECACC. The cells were cultivated in MEM medium supplemented with 10% fetal bovine serum and 1% PSN (penicillin, streptomycin, neomycin) in a humid atmosphere at 370C and 5% CO_2_. Briefly, the samples (0.5 X 0.5 cm^2^) were kept in fresh MEM medium for 24 hours. Then this culture medium was transferred onto a cell culture seeded the previous day (3 x 104 cells/well) and left 24 or 48 hours respectively.
- Neutral red (NR) assay: After 24h and 48h respectively from cultivation, the culture medium was removed and the neutral red solution was added (50 microg/mL). After an incubation at 37 degrees C for 3h, the neutral red solution was removed and replaced with the fixative solution (CH_2_O:CaCl_2_ = 2.5:1), for 3–4 minutes. NR retained in the cells was dissolved by using a discoloring solution (acetic acid: ethanol: distilled water = 1:50:49) and the plates were shaken for 15 min. The absorbance at 540nm was measured by using a Tecan Sunrise plate reader. The results were reported as percent of control (untreated cells), considered as 100% viable cells.
- Lactate dehydrogenase (LDH) assay: Cellular supernatants and lysates (50 microL each) were individually incubated with 50 microL mixed reaction solutions (Promega, WI, USA) at room temperature, for 30 minutes, protected from light. These mixtures were measured spectrophotometricaly at 490nm by using a 96–well plate reader (Tecan Sunrise). The percent of viable cells (LDH retained in the cells) was determined by using the following formula:

**Figure 1:**
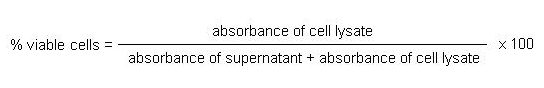
Formula for determining the percent of viable cells

Serum free culture medium (from medium replacements) was used as blank, which was deducted from all absorbance readings. The percentage of living cells corresponds to the percentage of cells that have not lost their membrane integrity and thus viability.
- Cell morphology: Cells grown in the presence scaffolds for 48h were fixed with cold methanol and Giemsa stained. The photomicrographs were taken by using a Zeiss Axio Observer D1 inverted microscope equipped with a camera (Carl–Zeiss, Germany).

## Results and discussion

### Properties of the obtained scaffolds

A scaffold used for tissue engineering requires a porous structure with a porosity not less than 70% and interconnected pores which allow cell growth and proliferation [12,13]. In this work, we have prepared four variants of collagen and collagen–agarose scaffolds, in the form of three–dimensional porous structure with heterogeneous pore size. The pore structure of these scaffolds was formed because of the freeze–drying technique used in their fabrication. Previous studies showed that the morphology of the pores is dependent upon the freezing temperature of the mixture before lyophilization [14]. In the present study, microporous structures were obtained when COL solution and COL–AG mixture were frozen at –40 degrees C

The porosity and density calculated for obtained scaffolds are presented in Table 1. 

**Table 1:** The porosity and density of scaffold variants

Sample	COL	COL–AG 2:1(A)	Col–AG 1:1(B)	Col–AG 1:2(C)
Porosity (%)	99.15	98.89	98.58	98.35
Density (g/cm^3^)	0.0272	0.0290	0.0350	0.0428

The high porosity value (99.15%) was obtained for COL scaffold. Incorporation of AG into the COL scaffolds slightly decreased its porosity, but the porosity was higher than 98% in all variants. Scaffold density values ranged between 0.0272 and 0.0428 g/cm^3^, indicating that the mechanical strength increases with the increase of the AG amount in the sponge

**Figure 2:**
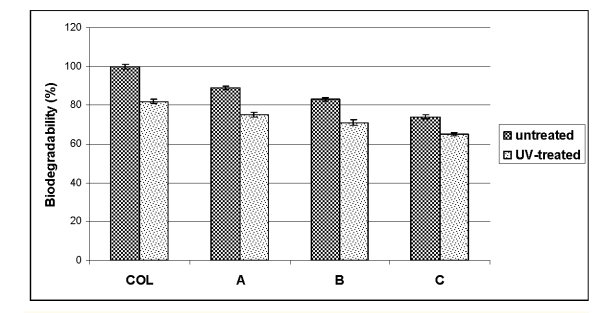
Degradation of COL based sponges after collagenase treatment. The results are mean for three determination + or - S.D.

Previous studies suggested that scaffolds used for tissue engineering should provide in the appropriate environment for cell proliferation and function and, in the same time, should be biodegradable [15]. Collagenase digestion can represent an in vitro measure of degradation rate for a biological implant. UV–untreated and UV–treated COL and COL–AG scaffolds were analyzed by collagenase digestion. The degraded collagen quantity was smaller for all UV–treated sample variants than for the UV–untreated ones (Figure 2). On the other hand, it was observed that AG content slightly influenced the biodegradability of UV–treated samples.

Our studies demonstrated that material exposure to UV increased the resistance of COL and scaffolds to enzymatic digestion. This result is supported by studies, which show that COL fiber can be cross–linked by UV radiation. UV exposure produces radicals from the nuclei of aromatic residues, such as those in tyrosine and phenylalanine and the binding of these radicals' results in the observed cross–linking [16]. These cross–links may inhibit the action of collagenase upon COL–based scaffolds and reduce their solubility.

### In vitro biocompatibility

**Figure 3:**
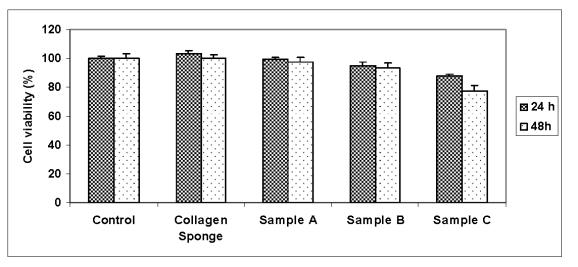
Neutral Red uptake viability assay of the studied samples. Results are mean of three–determination + or - S.D.

Biocompatibility of porous collagen scaffolds was evaluated qualitatively and quantitatively, according to current European standards (ISO 10993–5/2003). The viability of fibroblasts cultivated in the presence of COL based scaffolds was in vitro evaluated by measuring the NR uptake from viable cells and LDH retained in cells.The cell viability values after 24h and 48h from cultivation are shown in figure 2 and 3. The NR assay indicated a varied cell viability in the range 87.5–103.5% after 24h from cultivation and 77.2–100.3% after 48h respectively (Figure 3). The highest value of fibroblast viability was observed for COL–AG (2:1) the scaffolds and the lowest for COL–AG (1:2) the variant. 

**Figure 4:**
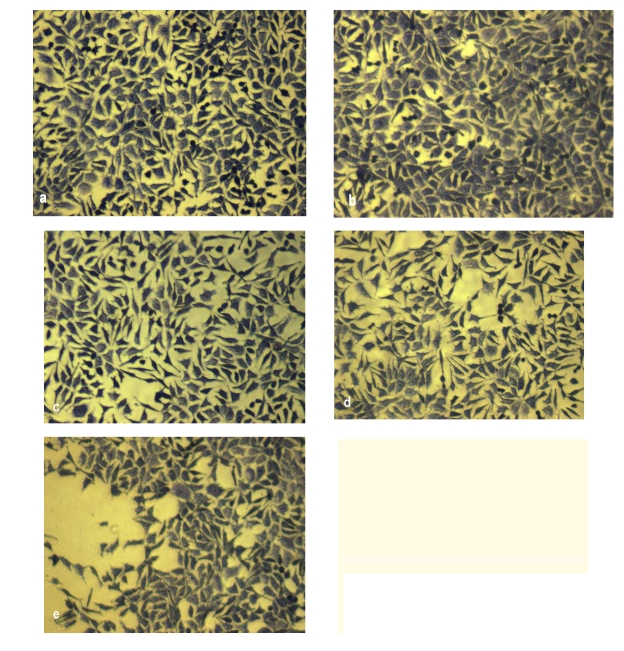
LDH viability assay of the studied samples. Results are mean of three–determination ±S.D.

The LDH retained in cells was evaluated in the same experimental conditions in which cells were analyzed by NR test. After 24h and 48h from cultivation, the viability of fibroblasts cultivated in the presence of COL and COL–AG variants A and B was higher than 85% in comparison with control sample, considered to be 100% viable cells. The samples containing a higher quantity of AG (variant C) induced a decrease of cell viability and a slightly toxicity respectively (Figure 4). 

**Figure 5:**
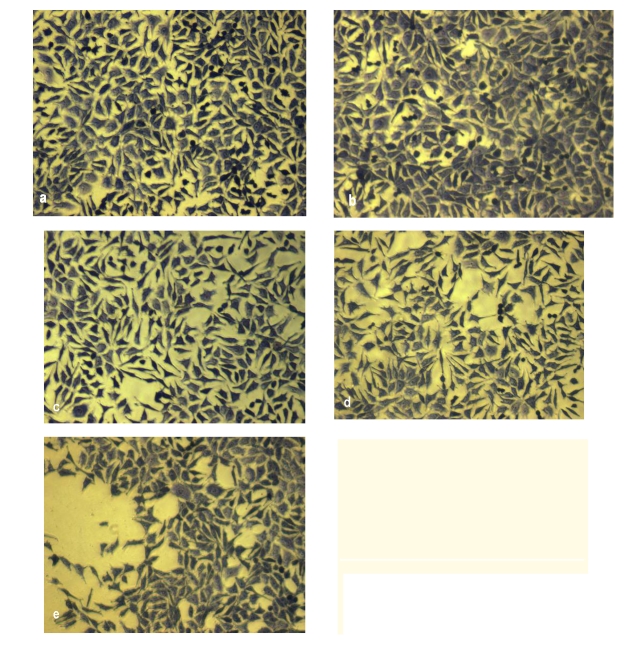
NCTC control cell  culture(a) and cells exposed for 48 h to the COL sponge (b), COL–AG 2:1 sponge (c), COL–AG 1:1 sponge (d), COL–AG 1:2 (e).

The morphology and proliferation of fibroblasts grown in the presence of studied scaffolds were evaluated after 48h from cultivation by light microscopy. The control cells cultured on plastic showed a normal fibroblast phenotype presenting euchromatic nuclei with 1–2 nucleoli and a clear cytoplasm (Figure 5). The analysis of fibroblast behavior in the presence of samples showed that normal cell morphology was maintained only in the case of COL and COL–AG (variant A and B) scaffolds (Figure 5). On the other hand, the proliferation rate of cells for these variants was similar to the control cells.

Variant C (COL–AG 1:2), induced a lower rate of cell proliferation and a change in the normal cell phenotype, inducing a granular cytoplasm and a higher number of intracytoplasmic vacuoles (Figure 5).

In conclusion, both cell morphological observations and viability test results demonstrated a higher biocompatibility of COL and COL–AG scaffolds, variants A and B.  Further in vivo studies are necessary to confirm the usefulness of COL–AG porous sponges for skin tissue engineering.
